# Demonstration of TGF-β and XIII_α_ in Endocardial Biopsies of Carcinoid Heart Disease Patients: an Immunofluorescence Study

**DOI:** 10.4021/cr48w

**Published:** 2011-05-20

**Authors:** Dorgrit Diepholz, Andreas Wilke, Bernhard Maisch, Dietmar Steverding

**Affiliations:** a Internal Medicine – Cardiology, Philipps University, Marburg, Germany; b Group Practice for Cardiology Papenburg, Papenburg, Germany; c Norwich Medical School, University of East Anglia, Norwich, U.K.

**Keywords:** Carcinoid heart disease, Fibrin-stabilizing factor, Transforming growth factor-β

## Abstract

**Background:**

Serotonin and other vasoactive substances play a critical role in the development of carcinoid heart disease, but the exact etiology of the illness is still unknown.

**Methods:**

By using immunofluorescence microscopy, we investigated the expression of transforming growth factor-β (TGF-β) and the presence of fibrin-stabilizing factor (XIII_α_) in endomyocardial biopsy specimens of patients with carcinoid heart disease. In addition, the tissue integrity of the specimens was studied by staining for laminin.

**Results:**

Both TGF-β and XIII_α_ co-localized in the endocardium beneath carcinoid plaques: while TGF-β was found within myocytes, XIII_α_ was detected on the surface of cells in fibrotic lesions stretching out into the tissue. Laminin staining revealed that the integrity of the endocardium was dissolved and that the tissue consisted of hypertrophic and hypotrophic myocytes.

**Conclusions:**

The results suggest that the presence of TGF-β and XIII_α_ in carcinoid heart lesions indicates that endocardial damage induced by serotonin and other vasoactive substances gives rise to an overshooting wound healing process.

## Introduction

Carcinoid tumors are rare neuroendocrine malignancies of the gastrointestinal tract and pancreas, and are derived from enterochromaffin cells [1]. These tumors produce and secrete peptide hormones and biogenic amines that can cause distinct clinical syndromes [2]. The clinical presentation of carcinoids, however, depends on the organ site of the tumor and whether the tumor is functioning or non-functioning. Only functioning tumors produce clinical symptoms as their secreted products are bioactive. The most common site of carcinoid tumors is the small intestine [2-5]. Small intestine carcinoids tend to metastasize to the liver. The bioactive substances produced by the liver metastases can easily reach the blood circulation and cause carcinoid syndrome. Between 18 - 50% of patients with carcinoid tumors develop carcinoid syndrome which is characterized by flushing, diarrhea and abdominal pain [1, 3]. Another characteristic of carcinoid tumors is their tendency to cause mesenteric fibrosis. If the fibrosis involves the endocardium of the right heart and the tricuspid and pulmonary valves, the associated condition is known as carcinoid heart disease. In about 10 - 50% of patients with carcinoid syndrome, carcinoid heart disease develops [1, 3].

The characteristic pathological findings in carcinoid heart disease are plaque-like deposits of fibrous tissue which are typically found on the endocardium of the right heart [4, 5]. Histologically, the plaques consist of myofibroblasts, smooth muscle cells and deposits of extracellular matrix, and are covered by an endocardial cell layer [6, 7]. Cusps and leaflets of the tricuspid and pulmonary valves are usually affected as well as the cardiac chamber, venae cavae, pulmonary artery and the coronary sinus [3, 5]. The fibrous deposits cause distortion of the affected valves that leads to stenosis and/or regurgitation [3]. The preferential right heart involvement is due to filtration of tumor products by the lung [8].

The exact pathogenesis of carcinoid heart disease is still unclear. The plaque formation within the heart has been linked to exposure to tumor-produced vasoactive substances, particularly to serotonin and tachykinins [8]. The pathophysiological role of serotonin is corroborated by the observation that the serotonin-releasing, appetite suppressant drug fenfluramine can cause valvular lesions similar to those seen in carcinoid heart disease [9]. In addition, transforming growth factor-β (TGF-β) was implicated in playing a role in the proliferation of fibroblasts and their matrix production in carcinoid heart disease [10].

In this study we investigated endomyocardial biopsies of seven patients with carcinoid heart disease by confocal immunofluorescence microscopy using antibodies against laminin, TGF-β and fibrin-stabilizing factor (FSF, XIII_α_).

## Materials and Methods

### Patient material

Cardiac tissue was obtained from seven patients by right ventricular endomyocardial biopsy during cardiac catheter examination. All patients were diagnosed with clinical symptoms of carcinoid heart disease. All subjects gave informed consent before the study. Cryosections of 3 - 4 µm were placed on coated glass slides and fixed with acetone at -20 °C.

### Antibodies

Primary polyclonal rabbit antibodies against human fibrin-stabilizing factor (anti-XIII_α_), human transforming growth factor-β (anti-TGF-β) and human laminin (anti-Lam) were obtained from the Behringwerke (Marburg, Germany), Promega (Mannheim, Germany) and Dako (Hamburg, Germany), respectively. The antibodies were used at dilutions of 1 : 200 (anti-XIIIα) and 1 : 50 (anti-TGF-β, anti-Lam). Secondary goat anti-rabbit Cy2 and Cy3 antibodies were purchased from Dianova (Hamburg, Germany) and used at a dilution of 1 : 400.

### Confocal immunofluorescence microscopy

After blocking the cryosections with goat serum in PBS, they were incubated with primary antibody in dilution buffer (1% BSA in PBS) for 1 h at room temperature (anti-Lam, anti-XIII_α_) or over night at 4 °C (anti-TGF-β). After washing three times with PBS, sections were incubated with secondary antibody in dilution buffer. In double labelling experiments, primary and secondary antibodies were sequentially applied (1st primary antibody, 1st secondary antibody, 2nd primary antibody, 2nd secondary antibody). Nuclei were stained by treating sections with 0.002% 7-aminoactinomycin in PBS [11]. The slides were mounted in FluoPrep (BioMerieux, Nurtingen, Germany) with DABCO as anti-fade reagent. Sections were examined with a Leica TCS confocal laser scanning microscope and images were recorded using a CCD camera.

## Results

Right ventricular endomyocardial biopsies taken from seven patients with carcinoid heart disease during cardiac catheter examination were analyzed by confocal immunofluorescence microscopy using anti-Lam, anti-TGF-β and anti-XIII_α_ antibodies. All specimens gave similar reactions and representative cases are shown. Controls omitting the primary antibodies gave no specific staining.

**Figure 1:**
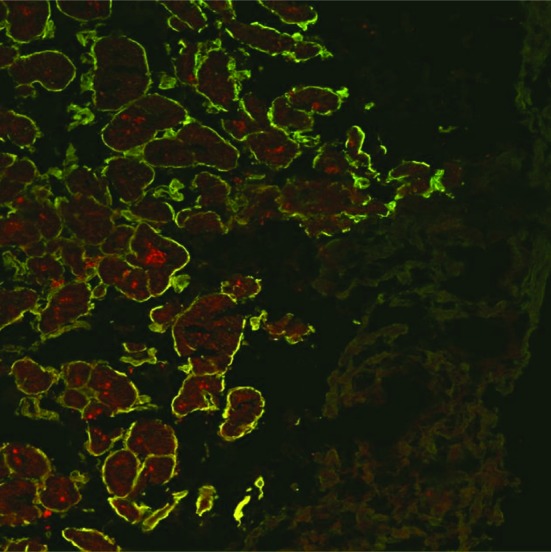
Confocal immunofluorescence microscopy of a biopsy specimen of a representative case of carcinoid heart disease. The cryosection was immunolabelled with rabbit anti-Lam and goat anti-rabbit Cy2 (green fluorescence) antibodies (magnification X 250).

At first, labelling experiments with anti-Lam antibodies were carried out on endocardial biopsy sections (Fig. 1). Specific immunostaining for laminin was found on the surface of myocytes. The myocytes themselves were recognized due to their auto-fluorescence. The immunostaining also showed clearly that the regular structure of the endocardium was dissolved and that hypertrophic and hypotrophic myocytes were present side by side in the tissue. The carcinoid plaque was not stained and formed a massive, weakly auto-fluorescent layer sitting upon the endocardium. The fine granular, red-colored structures seen within the myocytes were identified as lipofuscin deposits, an aging pigment found in the heart muscle [12].

**Figure 2:**
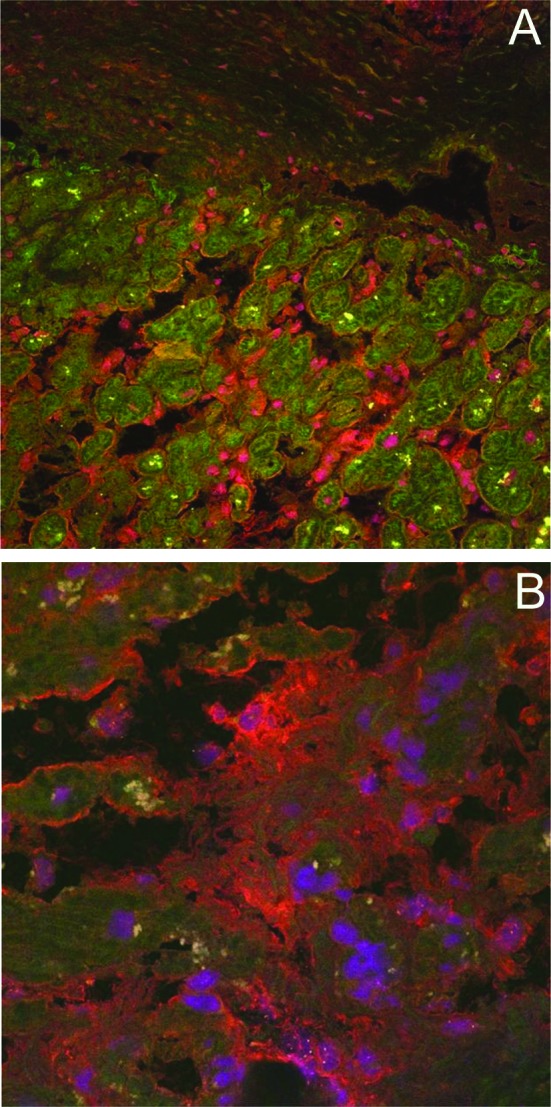
Double immunofluorescence microscopy of biopsy specimens of representative cases of carcinoid heart disease. Cryosections were double-immunolabelled with rabbit anti-TGF-β and goat anti-rabbit Cy2 (green fluorescence) antibodies and rabbit anti-XIII_α_ and goat anti-rabbit Cy3 (red fluoresecence) antibodies (magnification X 250 (A) and X 630 (B)). Nuclei were stained with 7-aminoactinomycin (purple fluorescence).

Next, double labelling experiments with anti-TGF-β and anti-XIII_α_ antibodies were performed (Fig. 2). Whereas immunostaining for TGF-β was detected in the cytoplasm of myocytes within the endocardium, the staining for XIII_α_ was restricted to the surface of cells located within fibrotic lesions stretching out into the tissue (Fig. 2A). The detection of TGF-β within myocytes is in agreement with previous findings of intracellular localization of TGF-β precursors in endocardial cells [10]. Again, the carcinoid plaque was recognized as a weakly stained, massive layer which contained only very few cells. Under the experimental conditions of double labelling, the lipofuscin pigments appear as yellow spots. At the edge of the fibrotic lesions, the surfaces of myocytes were stained positive for XIII_α_ (Fig. 2B). This finding indicates that XIII_α_ was deposited on the membrane of the myocytes.

## Discussion

In this study we provide evidence that TGF-β and XIII_α_, two proteins involved in the fibrotic healing process, may play a role in the pathogenesis of carcinoid heart disease. TGF-β and XIII_α_ occur in platelets and take part in the wound healing process. We found both proteins in fibrotic lesions of endocardial biopsies of patients.

Carcinoid heart disease usually develops in patients with carcinoid syndrome. In turn, carcinoid syndrome occurs in patients with enterochromaffin tumors which have metastasized to the liver. Enterochromaffin cells are the main neuroendocrine cell type occurring in the epithelia of the small intestine [1, 13]. They secret serotonin, tachykinins and other vasoactive substances in response to hormones, neural factors and acid [1]. In addition, more than 90% of the body’s serotonin is synthesized, stored and released by enterochromaffin cells [14, 15]. If liver metastases are present, the bioactive substances of enterochromaffin cells can easily spread throughout the body via the blood circulation causing carcinoid syndrome [2].

The exposure to vasoactive substances is believed to result in endocardial damage of the right heart [8]. Serotonin, on the other hand, has been shown to have a stimulating effect on subendocardial cell proliferation [16]. The potential pathogenic role of serotonin in carcinoid heart disease has been further underlined by animal experiments showing that long-term administration of serotoinin induced the formation of carcinoid-like plaques on cardiac valves [17]. Moreover, serotonin has been suggested to up-regulate the expression of TGF-β [18]. TGF-β has been previously implicated in the pathology of carcinoid heart disease [10]. In addition, TGF-β activates myofibroblasts and induces the synthesis of fibronectin and collagen [19], and thus accelerates the deposition of the extracellular matrix components and contributes to a permanent wound closure. In the present study we have shown that TGF-β co-localizes with the blood coagulation and wound healing protein XIII_α_ in fibrotic lesions of carcinoid heart disease. Although the main function of XIII_α_ is the cross-linking of fibrin molecules, it also takes part in wound healing and, as previous research indicated, in fibrotic processes [20, 21]. In this context it is noteworthy that fibrinogen and fibrin have been previously identified in carcinoid plaques [22].

The discovery of hypertrophic and hypotrophic myocytes detached from each other and stained for laminin is a clear sign that the tissue integrity of the endocardium is disturbed. Laminins are glycoproteins and components of the basal lamina [23]. They form independent networks and bind to cell membranes through integrin receptors and other non-integrin molecules. One function of laminins is the maintenance of the tissue integrity [24].

In conclusion, the detection of the wound-healing proteins TGF-β and XIII_α_ in carcinoid heart lesions suggests that endocardial damage caused by vasoactive substances leads to an overshooting wound healing reaction with persistent repair processes and, eventually, to a fibrotically altered heart.
